# From ‘Nerve Fiber Regeneration’ to ‘Functional Changes’ in the Human Brain—On the Paradigm-Shifting Work of the Experimental Physiologist Albrecht Bethe (1872–1954) in Frankfurt am Main

**DOI:** 10.3389/fnsys.2016.00006

**Published:** 2016-02-25

**Authors:** Frank W. Stahnisch

**Affiliations:** Alberta Medical Foundation/Hannah Professorship in the History of Medicine and Health Care, University of CalgaryCalgary, AB, Canada

**Keywords:** history of neuroscience, brain plasticity, Albrecht Bethe (1872–1954), Santiago Ramón y Cajal (1852–1934), neurorehabilitation, twentieth century, Germany, neurophysiology

## Abstract

Until the beginning 1930’s the traditional dogma that the human central nervous system (CNS) did not possess any abilities to adapt functionally to degenerative processes and external injuries loomed large in the field of the brain sciences (*Hirnforschung*). Cutting-edge neuroanatomists, such as the luminary Wilhelm Waldeyer (1836–1921) in Germany or the Nobel Prize laureate Santiago Ramón y Cajal (1852–1934) in Spain, debated any regenerative and thus “plastic” properties in the human brain. A renewed interest arose in the scientific community to investigate the pathologies and the healing processes in the human CNS after the return of the high number of brain injured war veterans from the fronts during and after the First World War (1914–1918). A leading research center in this area was the “Institute for the Scientific Study of the Effects of Brain Injuries,” which the neurologist Ludwig Edinger (1855–1918) had founded shortly before the war. This article specifically deals with the physiological research on nerve fiber plasticity by Albrecht Bethe (1872–1954) at the respective institute of the University of Frankfurt am Main. Bethe conducted here his paradigmatic experimental studies on the pathophysiological and clinical phenomena of peripheral and CNS regeneration.

## Introduction

Most neuroscientists at the beginning of the twentieth century held the opinion that the human brain lacked any functional capacities for repair, readaptation, and response to neuronal damage following degenerative diseases (e.g., Alzheimer’s Disease or Morbus Parkinson) and cerebral injuries (such as stroke; Stahnisch, 2003). Only a tiny minority of neurologists and neuropathologists sought to identify cerebral repair mechanisms that allowed the neurologically severed human brain to be seen in a different physiological light. To the fringe group of contemporary brain scientists (German: “*Hirnforscher*”) in an emerging interdisciplinary field—comprised of neurologists, psychiatrists, neuropathologists, and other allied scientists (Breidbach, 1996)—also belonged the neurophysiologist Bethe at the University of Frankfurt am Main (Anonymous, 1957). Based on his widely received contributions to the “Handbook of Normal and Pathological Physiology” (“*Handbuch der Normalen und Pathologischen Physiologie*,” 1925–1933) (Bethe, 1925a), which characterized him as a leader in the German-speaking neurosciences, Bethe became instrumental in the development of a new paradigm of “neuroplasticity” in the human brain (Bethe, 1925a). Together with his clinical colleagues, such as Kurt Goldstein (1878–1965) and the latter’s holist tradition in neurology at the Frankfurt Institute for the Scientific Study of the After-Effects of Brain Injuries (“*Institut fuer die Erforschung der Folgeerscheinungen von Hirnverletzungen*”) (Pow and Stahnisch, 2014), Bethe conducted pioneering research into several facets of neurorehabilitation and the use of prosthetics in brain-injured war veterans. Bethe’s neurophysiological research program laid some paradigmatic foundations for neuroscientific assumptions that are still used by modern-day scientists in the field of neurorehabilitation, neuroprosthetics, and neurotraumatology, such as “sprouting from axis cylinders” (“*Aussprossung aus den Achsenzylindern*”), “functional restitution” (“*Funktionsersatz*”), and “reconnection of the myelin sheaths” (“*Neurilemm-Naht*”) in the therapeutic approaches to nerve and brain injuries. By drawing on Bethe’s publications, archival materials from the Edinger Institute and the American Rockefeller Foundation, along with published letter exchanges between him and contemporary brain scientists, this article aims at characterizing some of the contributions of this exemplary and innovative neurophysiologist.

## Life and Work

**Figure 1:**
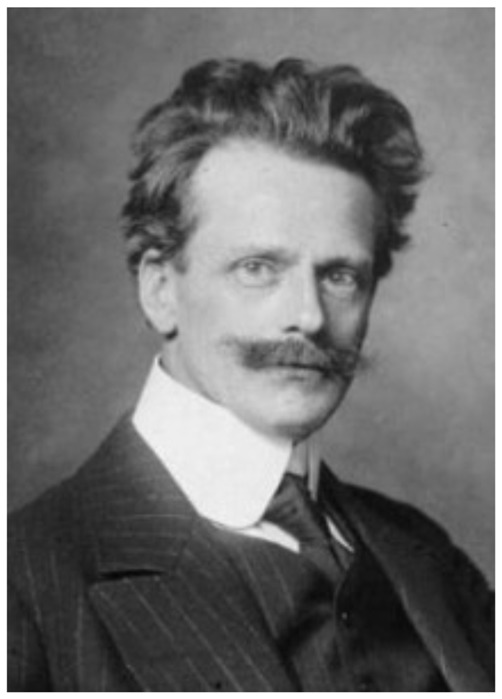
**Albrecht Theodor Julius Bethe (ca. 1910).** Photograph © courtesy of the Université Louis-Pasteur—Les collections de l’Institut d’anatomie pathologique de Strasbourg.

Albrecht Julius Theodor Bethe was born on April 25, 1872 in Stettin in Pomerania into a traditional family of doctors. The grandfather from his father’s side was a general practicioner in the city of Stettin; and the grandfather from his mother’s side later became a medical professor at the newly founded German University of Strasburg in the 1870’s (Gorzny, 1998). After his graduation from high school in his hometown, Albrecht Bethe continued to study medicine at the universities of Freiburg, Berlin, and Strasburg. He received his M.D. degree in 1895 at the Ludwig Maximilians University in Munich, with a thesis on the “Terminal Nerve Endings in the Frog’s Pharynx and Tongue” (“*Die Nervenendigungen am Gaumen und in der Zunge des Frosches*”). Soon thereafter Bethe was offered a position as an assistant professor (“*Wissenschaftlicher Assistent*”) at the newly founded Kaiser Wilhelms University in Strasburg, where he worked at the Physiological Institute under the leadership of physiologist Friedrich Goltz (1834–1902) (Schmidt-Boecking, 2007). This was also a time when the University of Strasburg witnessed an emergence of noticeable brain research activities that combined investigative approaches from anatomy, pathology, radiology, and surgery within a new interdisciplinary research field. During the Wilhelminian Empire’s restitution of this German-speaking university (1872–1918), the recently inaugurated Kaiser-Wilhelms-University was established to showcase the excellence of contemporary German post-secondary education. Moreover, the first two decades of the university’s existence, leading academics were hired for the medical faculty, including the anatomist Wilhelm Waldeyer, 1836–1921 (~development of the “neuron concept”), the pathologist Friedrich Daniel von Recklinghausen, 1833–1910 (discovery of “neurofibromatosis”), and the psychiatrist Richard von Krafft-Ebing, 1840–1902 (~pioneering studies of sexual psychopathology), who pushed the research envelope in their respective fields. These luminaries also counted among Bethe’s academic teachers (Figure 1) during his medical training from 1895 to 1911. In the year 1899, Bethe eventually submitted his second *Habilitation* thesis, entitled “Studies on the Central Nervous System of Carcinus Maenas” (“*Studien ueber das Centralnervensystem von Carcinus Maenas nebst Angaben ueber ein neues Verfahren der Methylenblaufixation*”) based on his innovative uses of methylene blue staining of central nervous tissue (Stahnisch, forthcoming).

During his tenure as a *Privatdozent* in experimental physiology in Strasburg, Bethe also met his later wife Anna (1876–1966), who was a descendant of wine farmers and merchants in Rhine-Hesse. Anna Bethe was a gifted musician in her own right and also found success as a literary author (Killy, 1999). Their son Hans Bethe (1906–2005), also born during the Strasburg period, would later rise to be a Nobel Prize Laureate in physics in 1967 and had been one of the leading specialists in the field of quantum mechanics in his American exile (Bernstein, 2006).

The brain research traditions cultivated at the University of Strasburg were of particular importance for Albrecht Bethe’s own work, especially the laboratory work of Friedrich Goltz (1834–1902) between 1872 and 1900/01 at the Institute for Experimental Physiology. Goltz’s investigations of functional brain and spinal cord localization received international attention in the scientific community. His acceptance was pointed out by the neuroanatomist Ludwig Edinger (1855–1918) in his autobiographical memoirs:

“Physiology is taught by Friedrich Goltz, whose research on the spinal cord has received a lot of attention, since he could show that many of the ‘higher mental functions’ are in fact the actions of specific and isolated parts of the nervous system” (Baeumer, 1996).

The neurophysiologist Ernst Julius Richard Ewald (1855–1921) was counted among the influential pupils and collaborators of Goltz from 1901 to 1917/1918, before Ewald received a professorial job at the Friedrich Wilhelms University in Berlin (Bethe, 1921). He was later hired back as one of Goltz’s successors, in order to continue his important auditory physiological work on the neurotransmission in the human auditory system in Strasburg (Trincker, 1959). Bethe similarly belonged to Goltz’s cohort of pupils in Strasburg, where Bethe became an adjunct professor (“*Privatdozent*”) of physiology following his second dissertation. He had been recognized as a rising talent in contemporary brain research, based on his (Bethe, 1903) neurophysiological textbook publication of *Anatomie und Physiologie des Nervensystems* which concisely summarized his earlier studies on cerebral and spinal cord nervous system localization—particularly his work on nerve ganglia changes after injuries (“*Veraenderungen der Ganglienzellen nach Trauma*”), retrograde never cell disintegration (“*retrograder Nervenzerfall*”), and the recovery of disconnected nerves (“*Zusammenheilen durchschnittener Nerven*”) (Bethe, 1903). Based on Bethe’s research success with the medical faculty of the Kaiser Wilhelm University, he was made an adjunct institute professor (“*Ausserplanmaessiger Professor*”) of neurophysiology.

Yet Bethe left Strasburg in 1911 when he received a professorial position at the Christian Albrechts University of Kiel in Schleswig-Holstein. He taught general physiology and zoology for medical students and pursued physiological research on non-vertebrate animals. This work is summarized in a concise methodical article, which introduced the experimental practices and approaches in the laboratory physiology of non-vertrebrates and was entitled “Non Vertebrates—Physiological Methodology, Metabolism, and Energy Exchange” Bethe (1911). Bethe stayed in Kiel for four years, when he eventually received a full professorship associated with the Neurological Institute in Frankfurt am Main in his specialization of neurophysiology (Fischer, 1957). Since 1918, Bethe also assumed the vital role as Editor-in-Chief of *Pflueger’s Archiv fuer die gesamte Physiologie* (now: the “European Journal of Physiology”) in his field of research (Bethe, 1918), which he held for almost two decades until 1937. During this productive period Bethe also published a second textbook on experimental physiology, entitled *Handbuch der normalen und pathologischen Physiologie* (Bethe, 1925b), which appeared in several editions until 1932.

However, once his wife was deemed “half Jewish” by the Nuremberg Race Laws of 1935 (Gruchmann, 1983), Bethe was ousted from his professorial position in 1937, as well as banned from taking up any university post again (“*Berufsverbot*”) (Brown and Lee, 2007). After the war ended with the unconditional surrender of the German Army on May-8, 1945 and new administrative and educational institutions were re-opened in the occupied zones, Bethe was fully rehabilitated as one of the first professors to serve at the re-opened West German universities. He was once again hired into the new Johann Wolfgang von Goethe University in Frankfurt am Main, while receiving his full academic rights and honors (Peiffer, 2004). For another two decades, he served as a university teacher and researcher in experimental physiology and as a founding member of the restored Frankfurt faculty of medicine. American politicians and Rockefeller Foundation officials struck a plan for Johann Wolfgang von Goethe University to emerge as a model academy for other zones in West Germany that should serve as landmark institution to serve the interests of the re-education and re-democratization programs for future elites (Rockefeller Archives, 1949). While influencing the fate of the West German Frankfurt University for another two decades and serving even as one of its early provosts (“*Rektoren*”), Bethe died from a severe heart problem at the age of 82 on October 19, 1954 in Frankfurt.

## Neurophysiological Research at the Neurological institute in Frankfurt Am Main

The period in which Bethe had joined the Neurological Institute—as an interdisciplinary institute for neurological, psychiatric, neuroanatomical, and neuropathological investigations—was characterized by profound transformations at the local university as well (Hammerstein, 2012). With the official foundation of a university in Frankfurt in 1914, Edinger as the chair of the Neurological Institute was made full professor of neurology and he received the assistance of two departments for basic and clinical research purposes: the department of anatomy and the department of pathology. These departments fulfilled service tasks for the adjacent city hospitals, such as post-mortem dissections of patients with previously difficult clinical symptoms, gross anatomical analyses of tumors, or the study of abortive embryos. Edinger could further rely on his own practice in the Frankfurt outpatient clinic for nervous diseases (*Frankfurter Poliklinik fuer Nervenkranke*). It merged into a significant clinical department in the communities adjacent to the medical campus in the Sachsenhausen neighborhood (Stahnisch, 2008). After the initial phase of university development came an expansion for the Neurological Institute, since Edinger was successful in luring Heidelberg neurologist Georg L. Dreyfus (1879–1957) to Frankfurt. Dreyfus joined him in his clinical efforts in the outpatient department during the First World War. He received here his *Habilitation* and was made an honorary affiliate professor with strong research interests in the pathologies of the CNS (Peiffer, 1998).

Although the Institute for Physiology was conceived of as an autonomous research institute in the newly founded university’s organizational hierarchy, the senate and provost nevertheless followed a proposal, which Edinger had submitted for the development and growth of the Neurological Institute. The proposal foresaw that the Institute of Animal Physiology in conjunction with the local Senckenberg Foundation should become an affiliated physiological research department with specific foci on neurophysiological research (Hoffmann and Stahnisch, 2014). Edinger was henceforth put on the search committee that would bring Bethe from the Christian Albrechts University of Kiel one year later, after the Prussian Ministry of Culture had approved the selection process (Klinke, 2006). Bethe possessed a very broad array of interests, particularly in the neurophysiology of nerve conduction, regeneration, and nutritional aspects of brain organization, which were summarized in his seminal work of the *Allgemeine Anatomie und Physiologie des Nervensystems* (Bethe, 1903). This book was a seminal introduction to neuroscientific research at the time, while it also included broad discussions of the elements of both reticular theory and contemporary evidence brought forward by the adherents of neuronist interpretations of nerve tissue organization. 

In fact, Bethe was one of the most significant representatives of the reticularist camp of neuroanatomists and neurophysiologists during the 1920’s and 1930’s. These researchers held that the elementary structure of the nervous system was built by a contiguous network of neuronal fibers (Lat. *reticulum*: network) instead of delineated histological elements, for which the neuronists as a competing group of contemporary brain researchers had advocated. Bethe himself followed particularly an electrophysiological interpretation of the neuronal network interaction, in that he claimed that nerve action potentials could only be transmitted in the nervous system, if they were conducted along continuing—and thus inseparated—nerve structures. Despite the fact, however, that by 1903 there were significant reports maintaining an individuality of neurons as discrete anatomical structures, such as the silver-staining advancements of later Nobel Prize laureate Santiago Ramón y Cajal (1852–1934) in Spain, the hematoxylin studies of neuroanatomist Albert Koelliker (1817–1905) in Wuerzburg, or the carmin red staining of nerve cells by Waldeyer in Berlin and Gustaf Retzius (1842–1919) in Lund, who particularly emphasized histological evidence from light microscopes for their views, Bethe departed from the views of this scientific camp. He rather understood the functioning of the human nervous system in terms of an electrophysiological necessity for the conduction of nerve potentials, along what he saw as a continuous network of interconnected neuronal elements.

The “reticulum idea” helped Bethe personally to explain electrical action potentials and transmission along the “neurilemms,” at a time when the structural morphology of the nerve “synapse” had not yet been fully explored and defined in light-microscopical terms (Bennett, 1999). In particular, Bethe was convinced that the neuronal elements of the nerve fibrils would provide a morphological substrate for electronic nerve conduction. It could enable and interconnect the cell membranes to build and sustain nerve cell to cell contacts in the brain and spinal cord. Nevertheless, this structural problem remained an issue of research contention and debate until the electron microscopic research contributions of the 1950’s and 1960’s (particularly: Palay, 1958), when then seemingly most neuroscientists accepted the neuron theory. At the time, however, it gives insight into Bethe’s use of contemporary cutting edge research techniques as well. His research would continuously intend to develop and methodologically advance these techniques over time (Bethe, 1926). Bethe’s use of new staining and research techniques fully represented his progressive and innovative character. His inclinations thereby brought him to consider “cerebral plasticity” at an early stage of his experimental career. Already in his Strasburg period, Bethe had considered the observed plastic behaviors in nerve cells to be a new research paradigm in the contemporary neurosciences. He applied this paradigm to both pathological as well as regenerative and adaptive situations of the human CNS.

**Figure 2:**
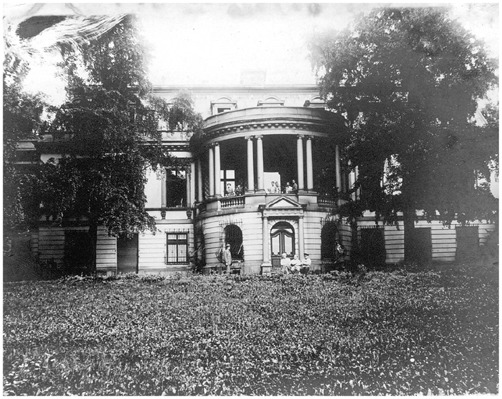
***The Villa Sommerhoff* was an encompassing sanatorium with rehabilitation facilities, medical services, and administrative buildings.** See Goldstein (1919).

After Bethe had arrived in Frankfurt in 1915, the Neurological Institute was subsumed among the military medical activities. The external institute of *Villa Sommerhoff* (Figure 2) was no exception, since it had been approved by the university board of regents as a recent specialized academic unit for neuropsychology and neuropathology, with the clinical assistant professor Walter Riese (1890–1974) becoming its deputy director and the institute remained in operation until the Nazis seized power in 1933 (Stahnisch and Pow, 2014). Bethe’s collaborator Goldstein later described the research context of the clinical program in Frankfurt in the following words:

“It consisted of a ward for medical and orthopedic treatment, a physiological and psychological laboratory for special examination of the patients, and theoretical interpretation of the observed phenomena, a school of retraining on the basis of the results of this research, and finally workshops in which the patient’s aptitude for special occupations was tested and where he was taught an occupation in line with his ability” (Goldstein, 1967).

Bethe had himself worked on neurosurgical problems of functional nerve sutures at the beginning of the war, research that he furthered through detailed animal experiments during the interwar period, by for example severing the spinal cords of cats at different levels, cutting longitudinally into the cortices of rabbits, and reconnecting peripheral nerves in dogs to varying superficial muscles of experimental dogs. Based on these experiments, Bethe could show for example that physiological nerve tissue repairs were possible and suggested that these could lead to acceptable therapeutic outcomes in human patients in the future (Bethe, 1916b). His animal experiments were built on the paradigm of the surgical crossing of the ischiadic nerves in dogs, while he further experimented with the transplantation of ischiadic nerve parts from one puppy sibling to another one. In his transplantation experiments the nerve parts were exchanged between the legs of a single dog and also from one individual to another.

**Figure 3:**
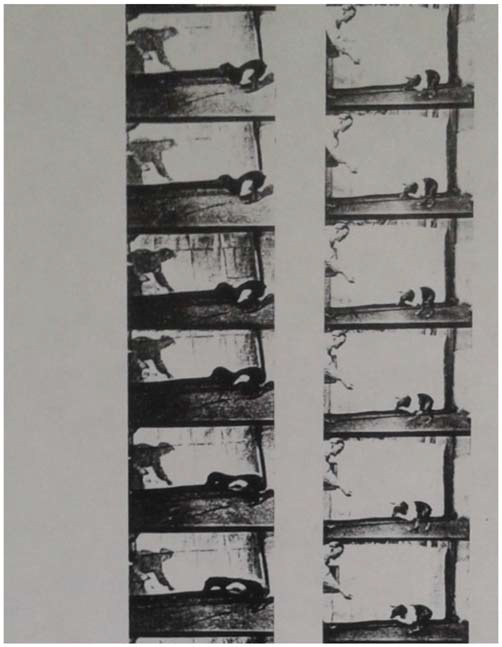
**Filmstill (separate images—origin ca. 1927), showing an experimental dog that was operated on the N. ischiadicus as a repair attempt.** On the left hand, Professor Albrecht Bethe is photographed, while handing out food to the dog to motivate it to move towards him. Reproduced in Bethe and Fischer’s (1931).

Bethe was able to show with these animal experiments that after a sufficiently long healing time (between several months and one-and-a-half years), the experimental dogs were again able to learn to walk in a nearly uninterrupted form. He interpreted this phenomenon from a physiological and functional standpoint, by pointing out that no constant and thus “irresponsive” neuronal connections could have been present. Had that been the case, then the function would have been irretrievably lost with the damaged neuronal tissue (as in clinical cases of stroke, cancer, or nerve injuries). Instead, the spinal cord apparently proved to be plastic and functionally adaptable, in that some of the original neurological functioning returned after the spinal injuries were treated (Figure 3). These research approaches were continued by Albrecht Bethe’s pupil Erich von Holst (1908–1962) in Munich, who later became a prominent German neuroethologist in his own right. Of course, a large amount of Bethe’s experimental research activities took place in the wider framework of military neurology in Frankfurt. This framework was greatly influenced by Kurt Goldstein, who succeeded Edinger after 1918 as the director of the Neurological Institute. Goldstein and his coworkers had kept close training and research ties to many of the nearby affiliated institutions during his time in Frankfurt from 1916 to 1930 (Stahnisch, 2014), while Bethe appreciated to have learnt from Goldstein’s group “that one part of the brain could learn the functions of the other.”

The time between 1910 and 1930 represented an era in the contemporary brain sciences which demonstrated increased investigations into de- and regeneration phenomena in several areas of the German-speaking morphological and physiological brain sciences (Stahnisch, 2009). This resulted in the appearance of a transformed concept of medical “trauma,” as it brought together hybrid notions and research practices from various disciplines (Bohleber, 2000). The field of neuroanatomy was particularly interested in finding the presumed microstructural lesions that all forms of trauma “imprinted” on the structure of the brain and spinal cord (Sulloway, 1979). This research focus became particularly visible in the laboratory experiments conducted on nerve fiber regeneration by Bethe, who for example varied numerous ligation experiments of peripheral and central nerves, while analyzing the de- and regeneration processes further with the application of osmium acid and chromic acid to visualize the peri-fibrillary connections and arrangements (“*Perifibrillensubstanz*”). Max Bielschowsky’s (1869–1940) research during this time, who referred to Bethe’s (1931) work repeatedly and was invited by the latter to also contribute to his *Handbuch der normalen und pathologischen Physiologie*, similarly focused on regeneration phenomena in CNS fibers (~nerve axons). With respect to his work on regeneration in the nervous system, he followed similar positions to those of Georges Marinesco (1863–1938) and Ion Minea (1878–1941) in Bucharest on peripheral nerve growth (1906), Giuseppe Levi (1872–1965) in Turin investigating sensory ganglion cell regeneration (1927), Santiago Ramón y Cajal and Jorge F. Tello (1880–1958) in Madrid on retinal regeneration phenomena (1934), who had all made pioneering contributions to the regeneration tradition in the modern neurosciences from their respective research directions (see further details in Stahnisch, 2003).

In his case, Bielschowsky pursued these analyses at another major center of the contemporary brain sciences within Oskar (1870–1959) and Cécile Vogt’s (1875–1962) brain research laboratory in downtown Berlin. This laboratory in the Prussian metropolis soon became too small and in the later 1920’s developed into the new Kaiser Wilhelm Institute for Brain Research on the northern outskirts of Berlin-Buch. While Bielschowsky closely collaborated with other researchers in the laboratory and institute, Wilder Penfield (1891–1976)—who had visited the institute during his sabbatical in Europe in 1928—nevertheless described the personal relationship between the Vogts and neuropathologist Bielschowsky as rather difficult. According to Penfield, Oskar Vogt had a tendency to intellectually “retract” and almost exclusively focus on the work with his wife Cécile:

“Oskar Vogt was all alone in one sense. He had every facility that he could use. He had associates, like his wife and the neurocytologist Max Bielschowsky, but no one to compete with him. When one is alone, it is too easy to go off on a tangent” (Penfield, 1977).

By 1927, Bielschowsky and Bethe no longer stood alone as neuromorphological research had progressed to focus more directly on microstructural changes of memory-related brain areas, such as the hippocampus, speech-related structural changes (as in the areas that the Breslau neuropsychiatrist Paul Broca, 1824–1880 and Carl Wernicke, 1848–1905 in Paris had discovered) or regenerative phenomena in the visual cortex—a process which added seeming credibility to the claims of veterans and the group of benevolent neurologists and psychiatrists. Hermann Oppenheim (1858–1919) had for a long time been the only prominent advocate who suggested the possibility of structural changes after war-related traumas (Radkau, 1998). But soon, researchers such as the neurohistologist Bielschowsky in Berlin (Bielschowsky, 1909), and Frankfurt neurophysiologist Bethe particularly addressed the regenerative phenomena of the brain (such as fiber sprouting, the filling of lost neuronal tissue, and vicarious functioning in the cerebral physiological centers) in their experimental laboratory investigations. At the same time, the search for a “psychopathic constitution” or psychiatrically relevant “degenerative dispositions” of the brain also continued in many clinical laboratories. During the early postwar period, it occurred to medical researchers that the neurological and psychiatric disorders seen in injured soldiers of the First World War were of an often changing nature, which made it necessary to find new concepts to accommodate the often drastic differences between soldiers who had experienced psychopathic episodes, while others returned home nearly unaffected by any medical conditions (Peiffer, 2004).

Bethe’s meticulous experimental physiological work was also attractive to Goldstein at Frankfurt University (Noppeney, 2001). This was due to Bethe’s intention to prove the general adaptability of animal and human bodies to their respective environments, as Jakob von Uexkuell (1864–1944) had introduced and as Theodor Beer (1866–1919), Bethe, and Uexkuell had published (in Beer et al., 1899). However, the historical scholarship has not pondered on the contributions which Goldstein’s school made to allied fields of contemporary neurology, including Gestalt psychology, functional philosophy, and brain psychiatry. These intellectual undercurrents could be found in the specific working settings at the *Institute for the Scientific Study of the After-Effects of Brain Injuries* in Frankfurt or later at the Clinical Neurological Department of the Moabit teaching hospital in Berlin. In terms of his work on the regenerative and plastic capacities of the human brain, particularly the collaboration with the experimentally oriented physiologist Bethe proved to be quite enriching for Goldstein. Bethe had almost exclusively devoted his research work to the experimental and clinical understanding of plastic neuronal processes, a working relationship that was further supported by the neuropathologist Karl Stern (1906–1975) and the experimental physiologist Adhémar Gelb (1887–1936) (Rothe, 1996):

“It was primarily through the close conjunction of Goldstein the neurologist with Gelb the psychologist that neuropsychology flourished in the vicinity of the Neurological Institute. The deep friendship between the two men is a testimony to their character. They were magnificently complementary in training and temperament, each capable of transmitting to the other much of his special skill” (Goldstein, 1967).

**Figure 4:**
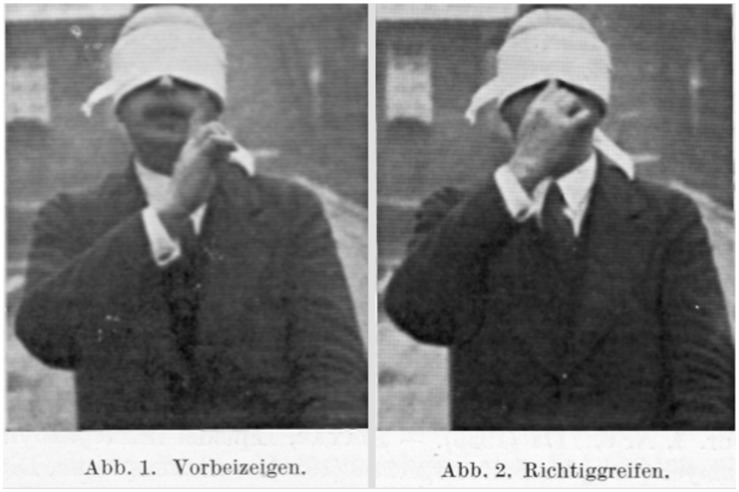
**A brain injured patient, who was studied by Bethe, Goldstein, and Gelb, is asked to pursue intentional movements to assess his lesions in the cerebellar cortex and the process of regeneration and functional gain following to a tight rehabilitation program.** In Goldstein and Scheerer (1941).

However, during the interwar period Goldstein, Bethe, and Bielschowsky felt prepared to take on the problem in a scientific and experimental way (Bethe, 1928a). The *Institute for the Scientific Study of the After-Effects of Brain Injuries* in Frankfurt am Main took a lead in this endeavor of researching the anatomical and physiological underpinnings of traumatic and psychological changes in brain-injured patients, while at the same time focusing on early approaches of neurorehabilitation in the quick reintegration of the neurological patients into new work environments (Bethe, 1916a). Also Bethe became very interested in this approach, since he saw in it a major chance of developing his basic physiological research program further into a more encompassing clinical physiological one (Figure 4). In this context, the Goldstein-Bethe collaboration developed further into a scientific study of early kinesiological viewpoints and human neurorehabilitation and prosthetics. Bethe described this process as a “turning point towards a new scientific study of energetics” that moved very closely in the direction of modern sport and rehabilitation science (Bethe, 1927, 1929c).

The collaboration between Goldstein’s neurological group and the experimental physiologist Albrecht Bethe was furthered by the love of both for technology and detailed experimentation. This is nicely illustrated by Bethe’s “*uses of the cylinder phonograph*” (“*Walzenphonograph*”), which had become a plausible instrument for analyzing speech through the invention of the American physicist and engineer Thomas Alva Edison (1847–1931) in 1877. The machine allowed for the recording of voices and Bethe immediately ceased on this opportunity to test Goldstein’s neurological patients for speech recovery (“*Ausreifungsprozesse*”) after traumatic brain injuries. When comparing the voices of neurological patients under rehabilitation conditions with those of normal test persons, Bethe was able to establish the chronology of behavioral and motor recovery processes—also in aphasic patients—and evaluating the impact of logotherapeutic and ergotherapeutic interventions in the clinical divisions of the Neurological Institute. This became later facilitated through the better availability of apparatuses that could easily be handled, such as when the Edison-Triumph machine which became available in 1905 (see also Bethe, 1934). Although it was initially developed to pass the time of the technophile physiology professor, this technique became an active research tool, recording the voices of Dr. Bethe and his patients and assistants within the institute. Bethe particularly liked to play around with vowels, and to record German sayings, jokes (“left of the stage there is a tree”), or dictated texts. These recordings helped determine the effects of brain lesions on the language centers, speech recovery, and clinical neurological phenomena associated with left and right hemisphere laterality.

## Albrecht Bethe and His Research on Nervous De- and Regeneration

Bethe became interested in neuroscientific issues of nervous de- and regeneration early on in his career while still working in Goltz’s laboratory in Strasburg. After becoming an independent researcher at the University of Kiel, he further broadened his experimental work on neuroplasticity to include investigations on peripheral nerve injuries from both neurosurgical and neuropathological perspectives. The First World War had just broken out and knowledge about peripheral and central forms of neurosurgery, wound treatment, and neurorehabilitation was desperately needed to care for the war-injured soldiers (Stahnisch and Nitsch, 2002). The foundations for this work were summarized initially in an overview article, which Bethe published during his later years in Goltz’s experimental physiological laboratory in Strasburg. This article was titled “New Research on the Regeneration of Nervous Fibers” in the *Archives of Physiology*, a leading physiological research journal at the time that was produced and published out of the University of Bonn’s Physiological Institute. In this publication, Bethe took the heuristic view that regeneration processes in nerve fibers were limited to the peripheral nervous systems (PNS) of animals and humans and that most of these biological growth processes had to be seen as physiologically abortive and non-functional in nature (Bethe, 1907a).

Bethe’s work on neuronal plasticity had its experimental beginnings and influences through the general biological interests in adaptive and regenerative capacities of several organic systems. In particular, it was triggered by his research on marine animals that began during an earlier research stay at the Experimental Marine Station in Naples, Italy. It formed the basis of Bethe’s line of reasoning regarding plastic reactions in the nervous system as well, since he thought that the same physiological and adaptive principals would likewise apply in the brain and spinal cord. The first steps to his research program were made in his excellent work conducted with marine animals, which was published in his article “Permeability of the Surface of Marine Animals” that he submitted to the American “Journal of General Physiology”. It detailed very refined chemical, histological, and physiological measurement approaches to the adaptation of the organism (“*Koerperanpassung*”) to its changing external *milieu* (Bethe, 1930a). Bethe started this research from the opinion that the surfaces of aquatic animals were semi-permeable. This was an idea which had been introduced by the Italian marine biologist Filippo Bottazzi (1867–1941), who pursued research on fish, muscles, and starfish. In line with Bottazzi’s and other Italian experimental biologists’ findings, only water was capable of passing through the animals’ integument, while other elements were altogether held back. In very rare instances had observations been made that ions would penetrate into the body of the experimental animals furnished by chemical means. Generally, the previous scientific thinking relied strongly on investigations that had proven the activity of osmotic reactions as the sole chemical cause for this phenomenon. The osmotic pressure of most marine animals was seen as being almost the same of the chemical and physiological ocean milieu around the experimental sea animals. This observation was further tested by Bethe in that he inserted fish and amphibians into several distinct concentrations of salty seawater, while measuring the osmotic changes and blood pressure effects in the test animals’ body physiologies, only to find that it quickly adapted (“*sich anpassen*”) to the respective milieu into which they had been put (Bethe, 1929a). Although Bethe did not address this research primarily from a neurological perspective, it was nevertheless foundational for his views on brain plasticity altogether. It was likewise illustrative of his broader focus on adaptive frameworks of biological processes that govern adjustment functions and plastic behaviors generally as environmental response mechanisms of physiological organisms.

**Figure 5:**
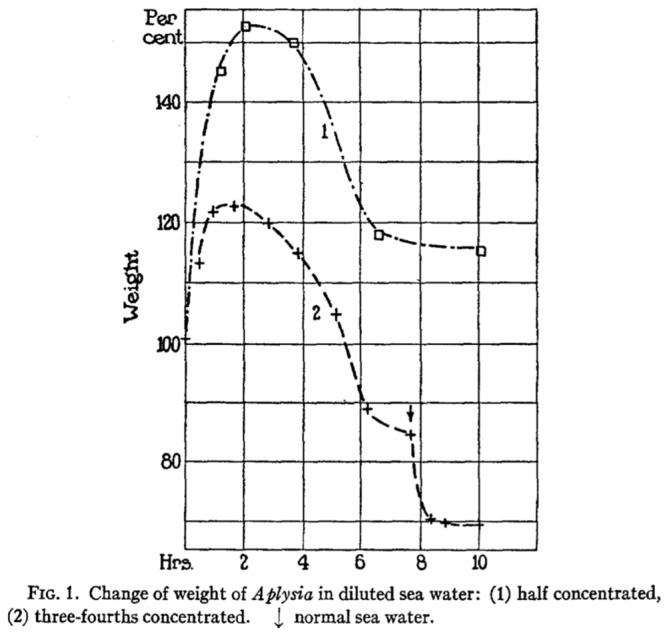
**Bethe’s (1930a) meticulous experimental research on the loss of weight in a sea slug (Aplysia) as a reflection of differing water salt levels**.

At the same time, according to the work of the Italian experimental physiologist and regeneration researcher Botazzi, whom Bethe referenced a lot in his laboratory work, it was evident that the size of fish, starfish, and muscles changed as a direct function of different seawater milieus. According to Bethe, this assumption could only be corroborated in experiments that would show that the organisms’ size levels corresponded directly as a function of ionic physiological pressures as well. However, the outcomes of the experiments differed, because the soft-skinned animals were covered with a mucous membrane. Bethe took Aplysia as his example—which also became a prominent test animal for neuroscientific memory research after the Second World War (Finger and Stein, 1982). Figure 5 is a representation of the weight differences in a sea slug following to its placement into seawater in different salt and other tonic solutions. Both curves detail the mean concentrations of ionic physiological fluids during different experimental series. Following to the sometimes-dramatic decreases in body weight, the sea animals’ blood became thickened to such an extent that it could hardly be extracted after cutting the skin open. This was a very different observation from the blood in normal animals, which was quite liquid and flowed out easily under traumatic conditions (Bethe, 1930a).

Bethe’s early research endeavors and theoretical discussions of neuronal plasticity (“*zentrale Regeneration von Protoplasmafortsaetzen*”) were based on his foregoing experimental work with marine animals. Taking what he saw as general and innate adaptive biological phenomena, he also developed his more specific neuroregeneration research in line with the prevailing thought of contemporary brain scientists, who discussed the possibility of adaptive and regenerative capacities in the human brain and spinal cord as well (Bethe, 1931). This productively departed from early morphological research on de- and regeneration phenomena since the end of the nineteenth century, which had almost exclusively concentrated on the PNS. It was easily accessible and controllable in laboratory research settings, such as in transplantation and regrowth experiments in ischiadic nerves of frogs and puppies or ablation experiments on optic nerves in goldfish (Clarke and Jacyna, 1987). Research with easily accessible peripheral parts of the nervous system emerged also as one of the major contributions, which historical scholarship attributed to the Spanish neurohistologist Ramón y Cajal. He had pointed out during the 1910’s and 1920’s that the CNS might also entail inherent regenerative and restorative capacities (Jones, 1999). This research in one way set forth the neuroscientific traditions of the previous century, in that it focused strongly on the anatomical properties of cerebral de- and regenerative processes. They had their origins in the physiological research on nervous outgrowth phenomena in dendrites by Johannes Mueller (1801–1858), advances in neurodevelopmental research, as pursued by Robert Remak (1815–1865), and the new cell pathology itself, following to the German morphological pathologist Rudolf Virchow (1821–1902), These perspectives originated in cell theory, in embryological investigations of nerve cell migration and in a crossover of physiological, embryological, and pathological methodology on morphological grounds (Borck, 1999).

Particularly at the beginning of the twentieth century many neuroanatomical researchers shifted their focus to the cellular properties of neuronal de- and regeneration phenomena, a development that laid the basis for a new tradition in the history of neuroplasticity that was also related to the creation of ever newer staining technologies for neurohistological work (DeFelipe and Jones, 1992). Staining technologies that had previously included chromic acid or methylene blue, were now added to by the new silver- and gold-derivatives form the Spanish and Italian schools of neurohistology (Bracegirdle, 1978). These new frontiers in the contemporary neurosciences gave rise to increasing research activities from a clinical perspective on the degenerative processes seen in neurosyphilitic patients, Multiple Sclerosis, and Alzheimer’s Disease. For others, the hippocampal system became the primary research area to study the morphological properties involved in adaptive processes of the brain and spinal cord (Bethe, 1895).

**Figure 6:**
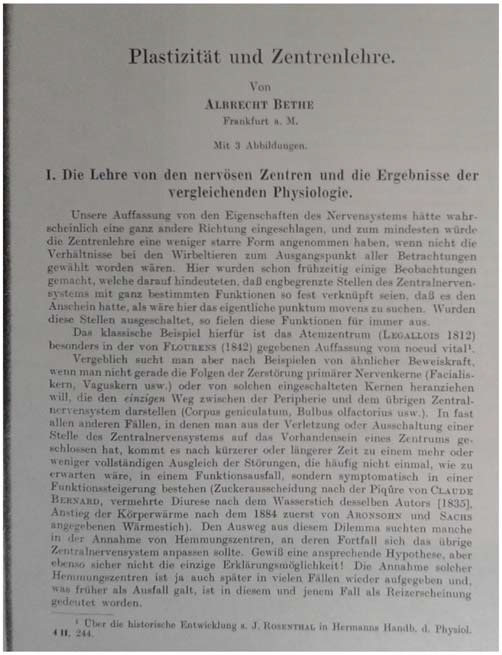
**Frontpage of Bethe’s article on “Plasticity and the Theory of Neuronal Localization, ” in Bethe and Fischer (1931)**.

Elements of the neurophysiological processes of neurodegeneration were specifically the ones investigated and revolutionized by Albrecht Bethe in his detailed and extended research program. Bethe’s program for example included anatomical, physiological, behavioral, and neuropathological information alike (Bethe, 1931, 1932, 1933). This led him to pursue regeneration (“*Regeneration*”) and neuronal plasticity (“*Neuronale Plastizitaet*”) throughout his experimental research in the Frankfurt institute, while interpreting sprouting phenomena, neurophysiological reactivation, and the behavioral relearning of previously lost functions, for example in locomotion, as positive capacities of animal and human nervous systems. In this sense, Bethe departed from some of the interpretations by the more morphologically oriented neuroanatomists, such as the work by the Spanish neurohistologist and Nobel Prize Laureate of Physiology or Medicine in 1906, Ramón y Cajal (1892a). Based on the organic systems he used in his research program over time, Ramón y Cajal tended to weigh the nature of regeneration phenomena in changing and different terms. In 1894, he developed early ideas about the new formation of axonal collaterals and dendrites in peripheral nerves (as positive forms of regeneration); in 1906, when researching spinal cord and cerebellar lesions, he interpreted the observed reactions as abortive reactions; in 1907, when working on ablation experiments in the cerebral cortex, he likewise viewed the sprouting phenomena under the microscope as pathological occurrences. It is around 1914 that Ramón y Cajal first used the term of “neuronal (adaptive) plasticity” to denote an innate capacity of the human nervous system to lead to regeneration in the cerebral pyramids and cerebellar peduncles. As a consequence of these changing experimental approaches, he emphasized the sprouting phenomena he had found in experimental dissections of nerve tracts in the cerebral cortex, the visual system, and the motor cortex in rabbits and cats. Yet it is also possible to find those other interpretations in Ramón y Cajal’s work, in which sprouting processes are perceived as abortive growth phenomena following to lesions rather than instances of the functional restitution in the injured CNS. Particularly his work on the hippocampus was thereby aligned with previous work on the dog cortex by Edward Albert Schaefer (1850–1935), the human pineal gland (in 1898) by Nicólas Achúcarro (1880–1919), and even later the research on sprouting in the human corpus ammonis (in 1934) of Rafael Lorente de Nó (1902–1990). This also formed the starting point of a number of major research publications that Albrecht Bethe had performed on the same subject (Figure 6), yet with a specifically physiological orientation on functional gain and neuronal restitution (Bethe, 1917, 1919, 1922).

**Figure 7:**
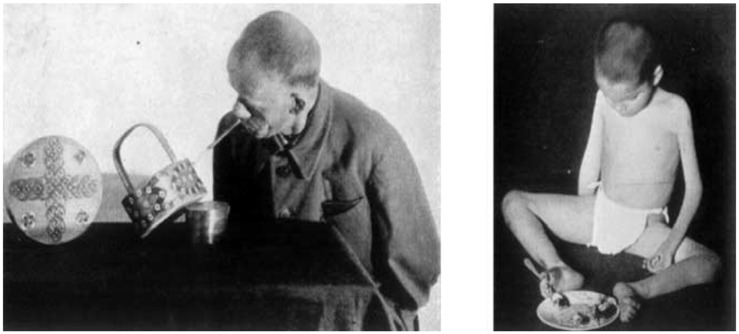
**(Left photograph)** A patient, who had lost both upper extremities and who was trained to paint with a pen in his mouth. **(Right photograph)** A child that was paralyzed and trained to eat with his legs (Bethe and Fischer, 1931), 1065.

However, it needed both the German (ed. 1929) and English (ed. 1991) versions of Ramón y Cajal’s later book on *De- and Regeneration in the Nervous System* (“*Degeneración y regeneración de los centros nerviosos*”) (Ramón y Cajal, 1908), that a new theoretical context and foundation for neuroplasticity research became available at the beginning of the twentieth century. As a consequence, in the year 1931, Bethe coined the notion of “plasticity” quite precisely in his “Textbook on Normal and Pathological Physiology” (“*Handbuch der normalen und pathologischen Physiologie*”) (Figure 7), yet surprisingly did not mention Ramón y Cajal in this particular publication—although citing him in many previous articles (e.g., Bethe, 1922). This work was also based on his earlier research on marine animals that he had pursued in the tradition of the notable Italian experimental physiologists, such as Filippo Botazzi and Eugenio Tanzi (1856–1934) (Bethe, 1907a).

Bethe described the plastic reactions in a physiological organism as its propensity to react (“*sich anpassen*”) to the varying external milieu in the contribution entitled “Plasticity and Localization Theory (“*Plastizitaet und Zentrenlehre*”) to his multi-authored textbook on experimental physiology (Bethe, 1931). His definition however remained rather broad and was flexible enough to constitute a productive and very successful boundary notion in the history of neuroscience (Jones, 2000). It is astonishing to find that Bethe did not receive Ramón y Cajal’s influential work on de- and regeneration in a more encompassing way, although clearly a lot of Cajal’s work had been translated into German and French by that time. It remained to Bethe’s co-worker, the experimental physiologist Ernst Fischer (1896–1981) (Engelhardt, 2002), that Cajal’s work was briefly received in a handbook chapter, entitled “The Adaptability (Plasticity) of the Nervous System” (“*Die Anpassungsfaehigkeit (Plastizitaet) des Nervensystems*”) (Bethe and Fischer, 1931).

Fischer mentioned the Spanish neuroscientist notion of nervous outgrowth from nerve stumps that had been previously experimentally cut, and in which the latter described the nerve axons’ “abortive reaction”—to form physiologically restored nerve functions again (in: “*Mecanismo de la regeneración de los nervios*”) (Ramón y Cajal, 1906). Likewise, Fischer referenced Ramón y Cajal’s concept of chemotaxis (in: “*La rétine des vertébrés*”) (Ramón y Cajal, 1892b) in this publication, while however focusing only on plastic processes seen in the PNS (Bethe and Fischer, 1931). That Bethe had substantially contributed to the emergence of the neuroscientific concept of “neuronal plasticity” was however only sporadically recognized in the scientific community. One such example was in an intriguing article that he published on “Plasticity of the Central Nervous System—A Neurosurgeon’s Experience of Cerebral Compensation and Decompensation” by the German neurosurgeon Hans Werner Pia (1921–1986) in Pia (1985).

The idea of “cerebral plasticity” did not have a lot of currency among contemporary brain researchers during the 1920’s and 1930’s. Following to Bethe’s work the attention of many German-speaking and German-trained *Hirnforscher* gradually came to change. The concept of normal neuronal plasticity, as postulated by Bethe in 1929, triggered research endeavors in many other areas of inherited pediatric brain disorders, in hemiatrophic, as well as neurodegenerative pathologies. As such, the notion of neuronal plasticity was reinterpreted as a central neuroscientific entity that related to a potential biological mechanism in recovery and readaptation after cerebral lesions.

**Figure 8:**
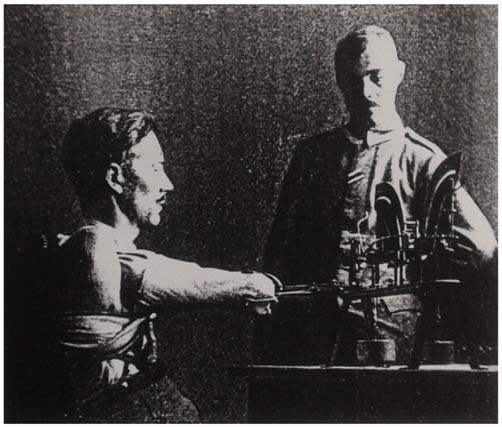
**A war veteran with an amputated stump above the elbow.** Following to a surgical adjustment of the remaining muscles to connect with two driving levers for an adjusted prosthesis, neurorehabilitation treatment commenced to train the movement and power of the remaining arm for later social and working purposes (Bethe and Fischer, 1931), 1111.

Bethe argued that an innate morphological substrate existed in the brain, which accounted for observable compensatory growth phenomena that he had even detected histologically after cerebral injuries or experimental lesions. This proposal, now widened to human neuroplasticity, was also reminiscent of his earlier work on cerebral laterality and cortical function distribution (Bethe, 1904b). Bethe had already discussed several major functional relations of plasticity related to structures in the animal and human brain, along with the central physiological regulation systems. He emphasized the amazing recovery and functional reorganization of the brain through innate plastic capacities and personal rehabilitation efforts over adequate periods of time (Figure 8). Bethe saw this recovery as an expression of the inherent functional development processes of the human brain (Bethe, 1905), which was however based on his foregoing experimental work in marine animals and smaller mammals.

The exceptional trajectory, however, of Albrecht Bethe’s experimental physiological work has recently received more attention in the scholarly literature. Bethe’s work has been traced from his early chemical and physical theories of nerve action and potentials (Bethe, 1904a) and unyielding research endeavors in the field of comparative anatomy and physiology (Bethe, 1897), to the debated area of “neuronal plasticity” and nervous repair mechanisms. German historian of medicine Florian Mildenberger, for example, has attributed Bethe’s interest in phenomena of biological adaptation, environmental complexity, and neovitalist functional research investigations, to his early collaborations with the systems biologists and evolutionary theorists Beer at the University of Vienna and Uexkuell at the University of Hamburg (Beer et al., 1899). Following to Mildenberger’s assessment, Bethe became increasingly interested in theoretical views of behaviorism and the study of psychological traits in animals and humans based on the particular morphological make-up of the CNS, which he saw as a means to objectify the general regenerative and adaptive processes of the organism through the modification of specific stimuli (“*Objektivierung der Anpassungreaktionen durch modifizierbare Reize*”). Bethe collaborated with both systems biologists in their 1899 paper. While several biological, psychological, and even sociological researchers were influenced by this publication and recognized it as a foundational piece for their own investigative activities, there was a noticeable retreat in the work of the three authors from their earlier views of behavioral adaptation. This retreat occurred despite the increasing attention that the notions of “adaptation” and “plasticity” received at the beginning of the last. Beer and Uexkuell however lamented that the somatic underpinnings of behavioral traits had not been understood enough to allow for a fully developed systems view of psychology and biological anthropology. These interchanges subsequently let to a withdrawal of one of the three authors—Theodor Beer—from active scientific work, and Jakob von Uexkuell in turn decided to review his own theoretical position. Yet instead of continuing more refined biological laboratory research, he rather delved new philosophical views over subjective vitalism and psychological behaviorist theories (Mildenberger, 2006). Despite the changes in the scientific positions of his coauthors, it appears that the publication and reception of their 1899 mutual paper had a different effect on Albrecht Bethe’s work. Indeed, increasingly Bethe strove to provide experimental evidence for his theoretical views on behaviorism, physiological adaptation, and neurological rehabilitation in his own research activities and publications (Bethe, 1901, 1906; Bethe and Woitas, 1926).

Of course, Bethe’s experimental investigations have to be seen against the background of Ramón y Cajal’s work on the neuron doctrine. Since the turn of the century, a revised version of the reticularism theory gained more and more academic currency among contemporary brain scientists (Frixione, 2009). It even appeared to replace the very focus on the “neuron” as the structural and functional unit that built up the human CNS. The revised theory was grounded in the detailed histological studies of neuronal cell morphologies by the Hungarian neuroanatomist and neurologist Stephan von Apáthy (1863–1922) (Bethe, 1923). Under this revised doctrine Bethe began to study the nervous fibrils (“*Nervenfibrillen*”) in ever more detail through several modified forms of experimental nerve lesions, which he performed in different levels of the nervous system in puppies and rabbits. He compared the nerve outgrowth and reconnection phenomena over time in very detailed quantitative and staining analyses, while assuming that the influence of electric currents was a stimulant of neuroregenration processes. Bethe himself came to the conclusion that action potentials would travel physiologically lengthways of the neurofibrils, which he understood as an interconnected reticulum made up of the individual neurons (Bethe, 1907b, 1910), as mentioned above.

In important ways, Bethe thus departed from Ramón y Cajal both methodologically and conceptually, where the Spanish neurohistologist had delved further into the task of rendering the new silver and gold staining methods useful for the study of de- and regenerative processes in neuronal structures. This work culminated in the redesigning of the “reduced silver nitrate method,” which the Italian neurohistologist Camillo Golgi (1843–1926) established as a most appropriate technique for analyzing the fine histology of the neurofibrils. Until 1904, Ramón y Cajal had produced more than twelve articles in Spanish, German, and French that described the neurofibrils of both invertebrate and vertebrate nervous systems. Ramón y Cajal examined these specimens under physiological circumstances (~ specimens from human brain dissections) as well as in experimental comparisons (~ by applying pathophysiological injuries to frogs, rabbits, and puppies as test animals), by also studying the role of the neurofibrils as “linear colonies” of cells in their contribution to nervous de- and regeneration. He summarized this work later in an overview article during the early 1930’s (Ramón y Cajal, 1933). This conclusion by Cajal would go on to trigger Bethe’s continued interests in the relationship between fibrils and cell morphology (Bethe, 1898/1899).

Hermann Stieve (1886–1956), who became an influential morphologist in the German Democratic Republic after the war, later characterized Ramón y Cajal (1934) work in his laudation address to the German Academy of Science in East Germany. For Stieve, the work of Cajal was “testimony” for the position seeing neurons as an important catalyzer for plastic processes (“*Sprossbildung*”) in the brain and spinal cord (Stieve, 1952), while he rather marginalized those experiments on animal cortices and visual neurological systems that preceding US or Spanish neurohistologists had provided, such as those of Elizabeth Hopkins Dunn (1867–1929), or Jorge Francisco Tello (1880–1958) (Dunn, 1917; Tello, 1911). However the vacillating views of Ramón y Cajal on plastic reactions in the brain, as outlined above, impeded a more quickly take-up of the latter’s publications. This was also visible, for example, in the poor reception of his studies on nervous de- and regeneration in Italy and France, where Aldo Perroncito (1882–1929) as well as Georges Marinesco (1863–1938) seemed to have accepted most of Ramón y Cajal’s publications (Clemente, 1964). Moreover, the Spaniard’s previous articles and books hindered a stronger adoption of his neuroregeneration work. Ramón y Cajal’s concept of cerebral and neuronal plasticity was hereafter unduly delayed in its application to contemporary experimental systems. Particularly contemporary staining technologies were not able to show the arborization of the full neurohistological layers (Pollack, 1905):

“It is hardly necessary to note that the methods of the dissociation and section of preparations impregnated with osmic acid show clearly only the old medullated sheaths. As to the ordinary methods, much used by the partisans of polygenism [i.e., the reticularists], which consists in staining sections previously fixed in sublimate, osmio-chromic mixtures, formalin, etc., with basic or acid aniline dyes, they show distinctly only the cordons composed of [Theodor] Schwann’s [1810–1882] cells, within which one can only vaguely see embryonic [regenerative] axons. The method of Bethe for staining the neurofibrils within the new medullated fibers is limited in its use, inefficient, and inconsistent … . As to the method of Golgi, which we have tried, it does not stain medullated fibers and impregnates non-medullated fibers with great irregularity and inconsistency …” (Ramón y Cajal, 1928).

More refined staining technologies only became available during the second half of the nineteenth century, so that these could be used by brain morphologists in their histological investigations of neurons, cell-cell connections, as well as nerve dendrites (Breidbach, 1996). For example, the gold-method was developed by the neurohistologist Golgi in Italy during the year of 1873 and methylene blue staining in the year of 1880 by the German microbiologist Paul Ehrlich (1854–1915). Both were later used by Bethe himself in his early research endeavors on “neuronal plasticity” (Bethe, 1895). The new staining techniques however also gave rise to continued methodological discussions about the artificiality and objectivity of the microscopic findings made in nervous de- and regeneration research programs of the time (Pannese, 1996):

“[T]he quarrel between the partisans of continuity and the autogeneticists arose from the difference in methods used. We are convinced that if Bethe . . . before fixing his attitude on the question of regeneration had [been acquainted] with silver procedures, [and] above all with the formulas of reduced silver nitrate which makes it possible to obtain trans- parent, thick section, he would have defended the Wallerian thesis” (Ramón y Cajal, 1933).

In the times previous to the development of the new silver-derived nerve staining technologies, a histological identification of individual neurons and the full complexity of their anatomical structures was an activity and procedure that was only to be pursued by very experienced researchers that had previously worked and understood the limitations of using eosine-hemeatoxylin, carmine red, or myelin sheath stains for the analysis of brain and spinal cord structures (Shepherd, 1991). Much until the early 1930’s, the resulting debate about the neuronal or network basis of the nervous system, a dispute into which Albrecht Bethe would actively take part as well (Bethe, 1930b), writing for example:

“Also these new concepts, in which the nervous system has been increasingly integrated into the areas of higher functional activities—other than this had been in the past—, will (only) have supporting value (in a heuristic sense). Sooner or later will these concepts be welded and advanced into a real theory, while also having to give way to other ideas. How could it be different in an area of research that is so complicated as this one! Haven’t we seen that in other and more accessible areas of physiology, and even in chemistry and physics, the theoretical positions had to be adjusted to allow for the integration of new empirical facts?” (Bethe, 1931).

## Discussion

In the context of neurohistological and neurophysiological work during the first half of the twentieth-century, many Central European neuroscientists increasingly developed an interest in further understanding the morphological properties and processes that the CNS possessed to react to the influences of inherited degenerative disorders and destructive pathologies and injuries. The contemporary methodological and histological debates had been directed at numerous instances concerned with nerve growth processes, the orientation of axonal sprouting, and the plastic reactions to the destruction of nervous textures in the CNS. Brain researchers, particularly in Germany, Austria, Hungary, and Spain delved into analyzing these “plastic” behaviors to structural damages to the human brain and spinal cord. Accordingly, refined investigative programs were developed that tackled the histological set-up of the CNS in terms of an adaptive and responsive organ. The new research paradigm in twentieth century neuroscience has been intriguingly summarized by Ramón y Cajal, who stated in 1937:

“For the anatomist, the histologist and the embryologist, bound to the hard bench of ana- lysis, the building up of general principles is, besides, in obedience to logical tendencies and almost unrestrainable impulses. It is forced upon us, moreover, by the very mode of action of our thinking mechanism, which is essentially practical and purposive, and presents to us every day the problem of the mechanical causes and the utilitarian motives. A structural or morphological arrangement having been observed, there invariably rises in our minds the question: ‘What physiological or psychological service does it render the organism?”’ (Ramón y Cajal, 1937, posthum).

Cajal’s and others’ explorations of such “unrestrainable impulses” in the human CNS along with the research activities on “the hard bench of analysis,” also brought contemporary brain researchers to realize the limitations of the very research methodologies that they used on the benches of their experimental laboratories. However, just when the next generations of neurohistological techniques had become more unfailing and better resolution optical instruments had appeared in the brain research departments of the time, did it become possible to discern the plastic processes in neuroanatomical structures. This view can be found explored in a concise form in Stahnisch and Nitsch (2002).

Most of the experimental research pursued by Bethe was based on see urchins, fish, and frogs, before he compared his findings with higher animals. This process was in line with other early twentieth-century brain scientists who started their experiments in the PNS of lower animals before heading towards the CNS. The new neuroscientific paradigm could be found as put to work in the experimental research programs on retinal transplantations (Tello, 1911) as well as the lesioning in rabbits’ visual systems (Leoz Ortíın and Arcaute, 1914). However, the investigations were impeded by insufficient identifications of the arborization in brain and spinal cord neurons. This methodological limitation is also visible during the introduction and early use of electron microscopical research approaches in the area of neuronal de- and regeneration research (Rasmussen, 1997). Eventually, contemporary neuroscientists made an innovation out of this histological research limitation, when they integrated electron microscopical research with the previous introduction of silver- and gold-staining methods of the nerve arborizations (Clemente, 1964).

Finally, one may see a good representation of the productivity of this new paradigm in brain research in the publications of the Canadian experimental psychologist Donald Hebb (1904–1991), who combined physiological and behavioral studies in a most instructive and helpful way. During the 1940’s, he came to develop an extraordinary theory of information processing in the nerve synapses based on the previous work by Ramón y Cajal in Spain formulating for example that the nerve synapses would represent instances of Bielschowsky’s recognition that the regenerative properties were located primarily in the so-called end feet (“*Endfuesschen*”) of each individual nerve (Breidbach, 1996). Accordingly, neuroplasticity became a guiding Leitmotiv in neurohistological and neurophysiological research into the genetic and restorative capacities of the human nervous system since the end of the nineteenth century (Borck, 1999).

## Summary and Conclusions

Bethe’s line of reasoning on neuronal plasticity was based on the experimental research on marine animals, which had pursued at Italian marine laboratories before the First World War, and findings that he later developed in his empirical work on the cortex of rabbits and dogs as well as comparisons to human clinical and behavioral observations in Frankfurt, Germany. From Filippo Bottazzi in Italy, Bethe had obtained the notion of the organism’s “milieu” to which individual physiological adaptations could respond. When researching pathological and neurosurgical processes before and after the First World War, Bethe focused primarily on issues of “nerve fiber regeneration.” During the interwar period, and under the influence of Kurt Goldstein, Walter Riese and others in Frankfurt, Bethe became increasingly interested in aligning his experimental findings with the clinical and psychological research approaches that allowed him to further assess and measure (e.g., with the use of the “phonograph” and his application of the “normal film sequences” to elicit trajectories of clinical neurological recovery) the “functional changes” in the brain due to nerve injuries over time. This led to his paradigm shifting work that intended to explore the physiological, structural, and behavioral transformations of the normal and pathological brain tissue. It has arisen as one of the most important research directions followed by many brain researchers today (Jones, 2000; Klinke, 2006). However, the relatively new technical term of “neuronal plasticity”—as popularized by Albrecht Bethe in Germany and Ramón y Cajal in Spain—had at first been rather marginalized when the latter neuroscientist had passed away (Finger and Stein, 1982). Only about a generation later, during the mid- 1950’s, did a renewed interest in the concept of brain plasticity reemerge in the modern neurosciences. This was largely also the effect of an introduction of innovative neuromorphological visualization and staining technologies (Klinke, 1993). Today, the paradigm of brain plasticity has emerged as a major tradition in neurohistology as well as experimental physiology, when the “structural alterations of axons and dendrites” (Cotman and Nadler, 1978), “behavioral adaptations” (Rosenzweig and Bennett, 1996), and “synapse formation” (Martin et al., 2000) are investigated with anatomical, functional, and genetic means.

This article has pointed to the paradigmatic changes that Bethe’s work in early twentieth century neuroscience brought about (Bethe, 1904b). Military neurology interests largely triggered Bethe’s research along with the high number of the brain injured war veterans who returned from the fronts during and after the First World War. It was particularly at the “Institute for the Scientific Study of the Effects of Brain Injuries” at the University of Frankfurt am Main, where neurophysiologist Bethe conducted his paradigm-shifting experimental research to investigate phenomena of peripheral and CNS regeneration. In the latter third of his career, Bethe then also addressed more philosophical questions, such as the history of experimental physiology (Bethe, 1929b), issues of the reorganization of scientific education in the medical curriculum (Bethe, 1928b), and the epistemological foundations of medical brain research at the time (Bethe, 1928c, 1929c).

## Author Contributions

The author confirms being the sole contributor of this work and approved it for publication.

## Conflict of Interest Statement

The author declares that the research was conducted in the absence of any commercial or financial relationships that could be construed as a potential conflict of interest.
